# Transmembrane protein 106a activates mouse peritoneal macrophages via the MAPK and NF-κB signaling pathways

**DOI:** 10.1038/srep12461

**Published:** 2015-07-28

**Authors:** Hui Dai, Dong Xu, Jing Su, Jingyuan Jang, Yingyu Chen

**Affiliations:** 1 Key Laboratory of Medical Immunology, Ministry of Health, Peking University Health Science Center, Beijing, China; 2 Center for Human Disease Genomics, Peking University, Beijing, China.; 3 Department of Pathology, School of Basic Medical Sciences, Peking University Health Science Center, Beijing, China

## Abstract

The M1 and M2 states of macrophage are the two extremes of a physiologic/phenotypic continuum that is dynamically influenced by environmental signals. Molecular mechanism analysis indicated that they gain M1 and M2-related functions after encountering specific ligands in the tissue environment. Here, we first characterized the previously unknown immunobiological functions of mouse Tmem106a. This protein is abundantly expressed on the surface of mouse macrophages. Activation of Tmem106a by stimulation with anti-Tmem106a upregulated the expression of CD80, CD86, CD69 and MHC II on macrophage, and induced the release of TNF-α, IL-1β, IL-6, CCL2 and NO, but not IL-10. These effects were largely abrogated by pretreatment with siRNA against Tmem106a. Notably, anti-Tmem106a significantly increased iNOS production and phosphorylation of STAT1, and had no effect on the ARGINASE-1 or p-STAT6 level, indicating that anti-Tmem106a activated macrophages and polarized them into M1-like macrophages. Further analysis found that anti-Tmem106a stimulation increased phosphorylation of ERK-1/2, JNK, p38 MAPK, NF-κB p65 and IKKα/β, and promoted nuclear translocation of the cytosolic NF-κB p65 subunit. Collectively, these data suggest that mouse Tmem106a might be a new trigger of macrophage activation and have some influence toward the M1 state through the activation of the MAPKs and NF-κB pathway.

Monocytes and tissue macrophages provide both immediate defense against foreign agents and assist during the initiation and development of the adaptive immune response. Diversity and plasticity are hallmarks of these cells. They can rapidly change their function in response to local microenvironmental signals^1^. Macrophages may undergo classical M1 activation or alternative M2 activation^2^. These classes are best considered a continuum of functional states that encompass a broad range of macrophage phenotypes with interchangeable characteristics^3^.

The classically activated M1 macrophages are characterized by the expression of high levels of pro-inflammatory cytokines, reactive nitrogen and oxygen intermediates, and strong microbicidal and tumoricidal activity. Alternatively activated M2 macrophages are considered to be involved in anti-inflammatory activity, promotion of tissue remodeling, tumor progression, and to have immunoregulatory functions^2^,^4^,^5^,^6^. The activation of macrophages into M1- or M2-type is dictated by the cytokine milieu of the tissue microenvironment^7^.

The effector function of macrophages is controlled by specific triggering signals such as cytokines, glucocorticoids and catecholamines, which stimulate differentiation into M1 or M2 macrophages^2^,^8^,^9^,^10^,^11^. In addition, signals such as LPS, unmethylated CpG oligodeoxynucleotides, the phagocytosis of necrotic cells, and the triggering of specific toll like receptors (TLR) can also influence the effector phenotype of activated macrophages^12^,^13^. Finding and characterizing new trigger signals in controlling macrophage differentiation is a key approach to determining how macrophages behave in immune response.

The human *TMEM106A* gene on chromosome 17q21.31 encodes TMEM106A, a member of the TMEM106 family. Our previous research revealed that human TMEM106A is a type II membrane protein, which is localized to the plasma membrane. Loss or reduction of *TMEM106A* expression is associated with promoter region hypermethylation in gastric cancer (GC). Restoration of *TMEM106A* expression induced GC cell apoptosis and suppressed GC cell growth, suggesting that TMEM106A is a tumor suppressor in GC^14^. TMEM106B is a type-II integral membrane protein, localized in the late endosome and lysosome compartments and is regulated by lysosomal activities^15^,^16^. TMEM106B is associated with cognitive impairment in amyotrophic lateral sclerosis and in the pathological presentation of Alzheimer’s disease^17^,^18^,^19^. Human TMEM106C is a differentially expressed transcript in ankylosing spondylitis (AS)^20^, and porcine TMEM106C was a positional and functional candidate for arthrogryposis multiplex congenita (AMC)^21^.

The TMEM106A gene is conserved in human, chimpanzee, Rhesus monkey, dog, cow, mouse and rat. Mouse Tmem106a is located on chromosome 11 and its function has not yet been determined. The present study was undertaken to analyze the expression and immunobiological functions of Tmem106a. Results arising from this study may help us to better understand macrophage activation and functional regulation mechanisms, and provide useful clues about immune regulation.

## Results

### Bioinformatic analysis and expression profiles of mouse Tmem106a

**Figure 1:**
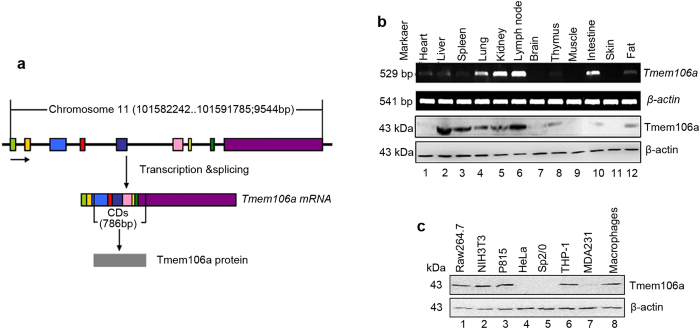
Mouse *Tmem106a* gene information and expression profile. (**a**) Schematic of the gene and mRNA structure of *Tmem106a*. The *Tmem106a* gene is located on chromosome 11, has nine exons and encodes a protein with 261 amino acid residues. (**b**) mRNA and protein levels of *Tmem106a* from various tissues in C57BL/6 mice were detected by semi-quantitative RT-PCR and Western blotting. (**c**) Protein expression of Tmem106a in mammalian cell lines and mouse peritoneal macrophages was detected by western blot. β-actin was used as the internal control. Complete electrophoretic gels and blots are shown in Supplementary Figure S4.

Transcription of the mouse *Tmem106a* gene, which is located on chromosome 11 and encompasses nine exons and eight introns, is shown in Fig. 1a. The full-length of mouse *Tmem106a* cDNA is 2301 base pairs. The ORF encodes a predicted protein of 261 amino acids with an isoelectric point of 7.04. The full-length cDNA and predicted amino acid sequences are shown in Figure S1. Alignment of *Tmem106a* sequences from various animals clearly demonstrates that *Tmem106a* is highly conserved (Figure S2 and Figure S3a). Transmembrane (TM) analysis (http://www.cbs.dtu.dk/services/TMHMM-2.0/)^22^ suggests that mouse Tmem106a is a type II transmembrane protein with a conserved TM domain (amino acids 93-115) (Figure S3b). To our knowledge, no functional studies have been performed on this protein.

The mRNA expression of Tmem106a was confirmed by semi-quantitative RT-PCR in a variety of normal mouse tissue samples. Figure 1b (upper panel) shows that high levels of *Tmem106a* mRNAs were observed in lung, kidney, intestine and lymphoid node. For subsequent experiments, we produced a rabbit anti-mouse Tmem106a polyclonal antibody using Tmem106a peptides (Figure S1, boxed sequences). This rabbit anti-mouse Tmem106a antibody was used to survey the expression and localization of Tmem106a protein. Consistent with the results of RT-PCR, the Tmem106a protein was detectable by Western blot in various mouse tissues (Fig. 1b, lower panel). The expression pattern of Tmem106a protein was also measured in various tumor cell lines and mouse peritoneal macrophages (Fig. 1c). Tmem106a protein was expressed in RAW264.7, NIH-3T3, P815, THP-1 and mouse peritoneal macrophages, but not in Sp2/0, HeLa or MDA231 cells.

### Tmem106a localizes on the cell surface of mouse macrophages

**Figure 2:**
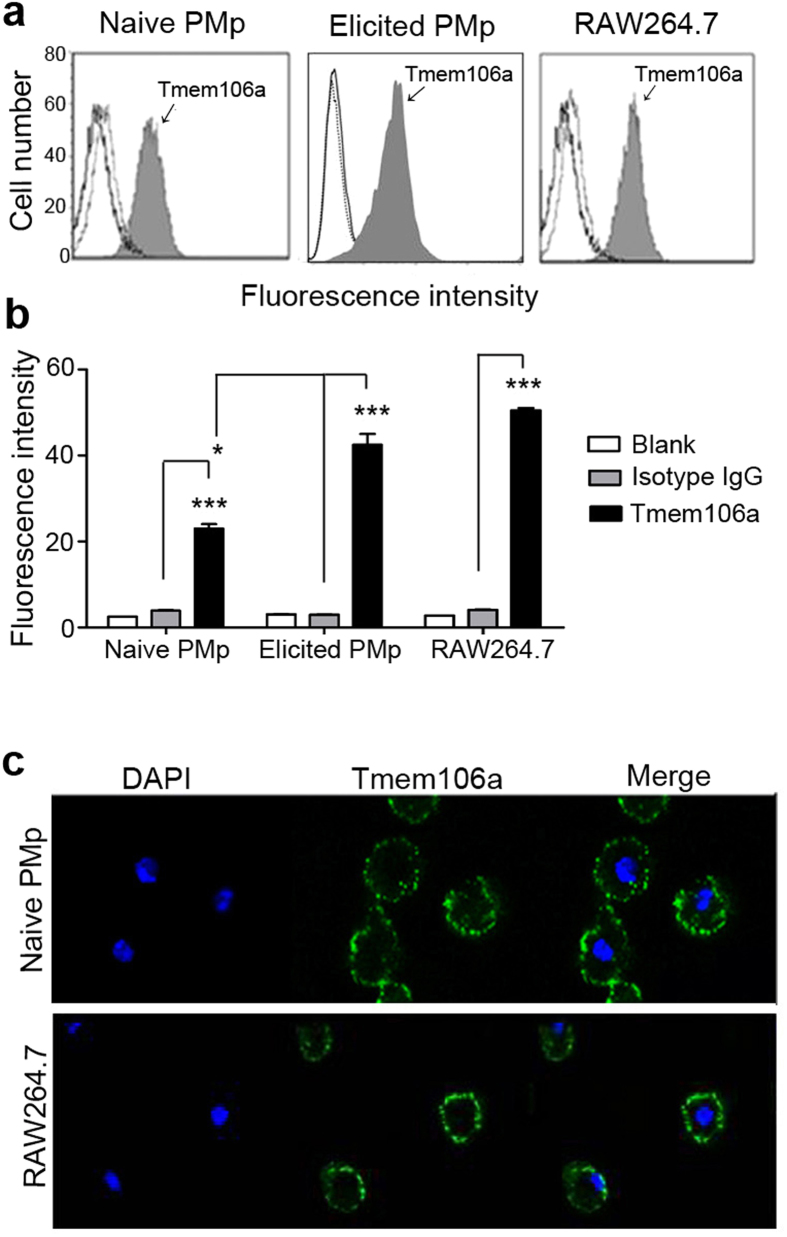
Tmem106a locates on the mouse macrophage surface. (**a**) Mouse naïve or thioglycollate-elicited peritoneal macrophages and mouse tumor line RAW264.7 were sequentially treated with anti-Tmem106a antibodies and FITC-labeled goat anti-rabbit IgG on ice, and then analyzed using a BD flow cytometer, counting 10,000 viable cells. The histograms are shown (filled histograms). Cells stained with isotype control antibodies (rabbit IgG) plus detection antibodies (dotted gray lines) or detection antibodies alone (solid lines) were included as specificity controls. CellQuest software was used for the analysis of the results. (**b)** The results are expressed as mean ± SD from three independent experiments (*p < 0.05, ***p < 0.001). (**c**) Viable naïve peritoneal macrophages (upper) or RAW264.7 cells (lower) on glass slides were treated with anti-Tmem106a antibodies followed by FITC-labeled goat anti-rabbit IgG and DAPI (for blue-staining of cell nuclei). The slides were then observed under a Leica confocal microscope.

The data above indicate that Tmem106a expressed in mouse macrophages. Bioinformatic prediction program (http://symatlas.gnf.org/SymAtlas/) predicts that the highest *Tmem106a* expression would occur in mouse macrophages. To further confirm this prediction, mouse macrophages, including thioglycollate-elicited cells, were freshly prepared from the abdominal cavity, stained with FITC coupled rabbit anti-mouse Tmem106a antibody or isotype IgG for 1 h at 4 °C, and then subjected to flow cytometric analysis. RAW264.7 cells were also stained by the same method. Each cell type mentioned above was positively stained by anti-mouse Tmem106a (Fig. 2a,b). Importantly, the fluorescence intensity of Tmem106a expression on thioglycollate-elicited macrophages was much more than that in naïve macrophages, suggesting that Tmem106a may be involved in the inflammation process. The stained macrophages were also observed under confocal laser scanning microscopy. Figure 2c shows that Tmem106a was expressed specifically on the peritoneal macrophage (PMp) and RAW274.6 cell surface, consistent with its character as a transmembrane protein predicted by bioinformatic analysis (Figure S3b).

### Anti-Tmem106a induces the activation of macrophages and M1 polarization

**Figure 3:**
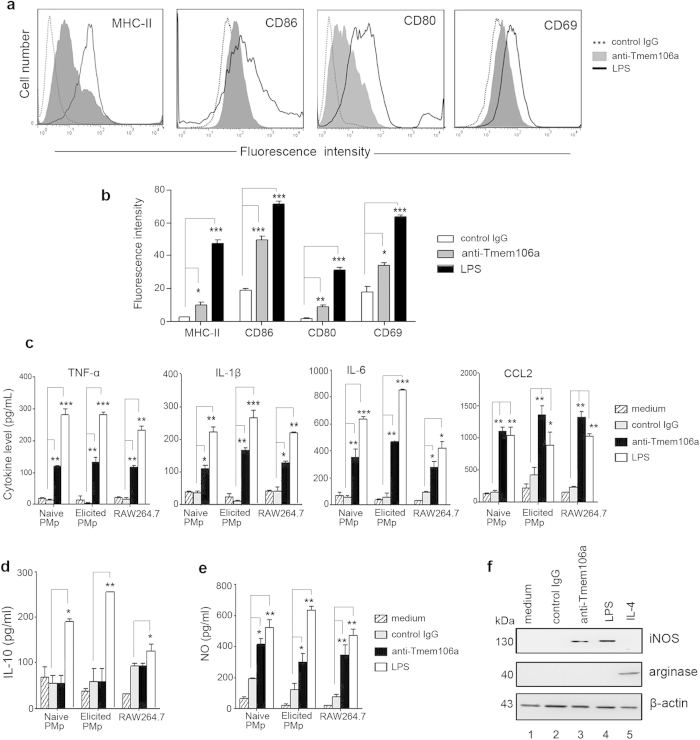
Effects of anti-Tmem106a on macrophage activation. **(a)** Freshly prepared mouse macrophages (4 × 10^6^ cells/well) were treated with isotype control IgG (dotted line), anti-Tmem106a (filled histograms) or LPS (solid black line) for 20 h. The cells were then stained with FITC-conjugated antibodies against murine MHC class II, CD86, CD80 and CD69, followed by flow cytometric analysis. Results are representatives of three independent experiments. (**b**) The results are expressed as mean ± SD from three independent experiments (*p < 0.05, **p < 0.01, ***p < 0.001). (**c**, **d** and **e**) Mouse macrophages from peritoneum (thioglycollate-elicited or not) and RAW264.7 were treated with isotype control IgG, anti-Tmem106a or LPS for 40 h. Concentrations of TNF-α, IL-1β, IL-6, CCL2, IL-10 and NO in the cultural supernatants were determined. The results are expressed as mean ± SD from three independent experiments (*p < 0.05, **p < 0.01, ***p < 0.001). (**f**) Mouse macrophages were treated with or without isotype control IgG, anti-Tmem106a, LPS or IL-4 (10 ng/ml) for 24 h. The levels of iNOS and arginase-1 were analyzed by western blot. Full-size images of western blots are shown in Supplementary Figure S5.

The expression of Tmem106a in mouse macrophages strongly suggests that Tmem106a may play an important role in macrophages. Using purified antibodies, we next assessed the biological activity of Tmem106a in macrophages. Phenotypic and functional maturation in macrophages were observed following anti-Tmem106a stimulation. Mouse peritoneal macrophages were treated with anti-Tmem106a (10 μg/mL), LPS (5 μg/mL) or isotype control IgG (10 μg/mL) for 20 h at 37 °C before staining with anti-mouse CD80, CD86, CD69 or MHC class II (Ia/Ie) for 1 h at 4 °C, followed by flow cytometric analysis. Compared with isotype IgG group, treatment with anti-Tmem106a led to the upregulation of CD80, CD86, CD69 and MHC class II in murine peritoneal macrophages, indicating that the macrophages had been activated (Fig. 3a,b). These effects were similar to, but to a lesser extent than, the effects observed following LPS treatment.

M1 and M2 macrophages all express CD80, CD86 and MHC-II molecules. We therefore next determined whether anti-Tmem106a induced the polarization of macrophages. M1 macrophages express high levels of pro-inflammatory cytokines such as IL-1β, TNF-α, IL-6 and chemokine CCL2 to intermediate strong microbicidal and tumoricidal activity. Whereas, M2-primed macrophages produce arginase-1, IL-10, and growth factors such as transform-ing growth factor-β (TGF-β) among others. LPS is often used as a positive control to induce the secretion of pro-inflammatory cytokines and promote M1 polarization. To analyze the effects of anti-Tmem106a on macrophage polarization, mouse macrophages from the peritoneum (thioglycollate-elicited or not) and RAW264.7 were cultured with or without anti-Tmem106a (10 μg/mL), isotype control IgG (10 μg/mL) or LPS (5 μg/mL). The levels of IL-1β, TNF-α, IL-6, CCL2 and IL-10 in the supernatant were determined. Figure 3c shows that, compared with isotype IgG, anti-Tmem106a obviously increased the production of TNF-α, IL-1β, IL-6 and chemokine CCL2 (Fig. 3c) in mouse PMp with or without thioglycollate treatment. These effects were similar to, but to a lesser extent than, the effects observed following LPS treatment. By contrast, the levels of inhibitory cytokine IL-10, which induces the M2 macrophage phenotype, did not change significantly in mouse PMp treated with anti-Tmem106a when compared with isotype IgG control group. Moreover, IL-10 level was lower in the anti-Tmem106a-treated group than in the LPS group (Fig. 3d). The effect of anti-Tmem106a on RAW264.7 cells was similar to that on PMp (Fig. 3c,d). The above data indicated that anti-Tmem106a treatment increased the sensitivity of mouse peritoneal macrophage; the cytokine secretion profile of the anti-Tmem106a-treated macrophages was consistent with the M1 phenotype.

We next examined the secretion of nitric oxide (NO) which is a hallmark of M1 macrophages, in the culture supernatant of mouse PMp using a nitrite detection method. Figure 3e shows that compared with the control IgG, anti-Tmem106a treatment significantly increased the secretion of NO in PMp and RAW264.7 cells; the effect was comparable to that of LPS. Taken together, these results indicate that anti-Tmem106a may induce the polarization of macrophages towards the M1 phenotype.

To further characterize the changes in macrophage polarization, we assessed the M1 marker iNOS as well as the arginase-1, which is an M2 marker. Peritoneal macrophages were incubated with or without control IgG, anti-mem106a, LPS or IL-4 for 24 h. In this experiment, IL-4 is used as a positive control to induce the expression of arginase-1 and promotes M2 polarization. Protein expression was assessed by western blot with antibodies specific for iNOS and arginase-1. As illustrated in Fig. 3f, compared with the IgG control, a high expression of iNOS was observed after stimulation with anti-Tmem106a. There was a similar result in the LPS positive control group. However, arginase-1 expression was not observed in either the anti-Tmem106a or LPS groups. As a positive control, arginase-1 protein was detected in IL-4-stimulated cells. Taken together, these results suggest that anti-Tmem106a induces the differentiation of M1 macrophages.

### Knockdown of *Tmem106a* attenuates the activation of macrophages induced by anti-Tmem106A

**Figure 4:**
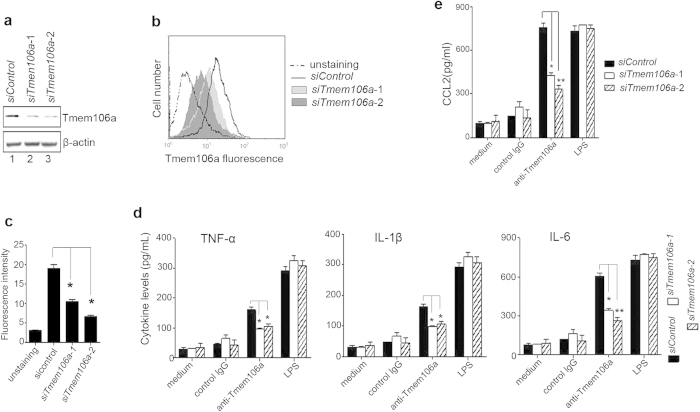
Knockdown of *Tmem106a* attenuates pro-inflammatory cytokine release induced by anti-Tmem106a. Mouse macrophages were transfected with siControl RNA or *siTmem106a* RNA for 24 h, followed by analysis of the expression of Tmem106a protein by (**a**) Western blot or (**b** and **c**) flow cytometry. Full-size images of western blots are shown in Supplementary Figure S6. (**d** and **e**) Mouse macrophages from peritoneum were pretreated with *siControl* or *siTmem106a-1/2* for 24 h, followed by incubation with isotype IgG control, anti-Tmem106a or LPS for 40 h. Concentrations of TNF-α, IL-1β, IL-6 and CCL2 in the cultural supernatants were determined using an ELISA kit. The results are expressed as mean ± SD from three independent experiments (*p < 0.05, **p < 0.01).

In order to elucidate the specificity of anti-Tmem106a antibody on Tmem106a in macrophage activation, further analysis was performed in *Tmem106a*-silenced macrophages. Using Western blotting and flow cytometry, we identified two siRNAs (*siTmem106a-1* and *siTmem106a-2*) effective against *Tmem106a* in mouse PMps (Fig. 4a–c). Mouse peritoneal macrophages were transfected with siRNA against *Tmem106a* for 24 h, then treated with control IgG, anti-mem106a or LPS. The levels of TNF-α, IL-1β, IL-6 and CCL2 in the culture supernatants were determined using ELISA kits. The results indicated that silencing of *Tmem106a* reduced the secretion of these pro-inflammatory cytokines induced by anti-Tmem106a (Fig. 4d,e). However, *Tmem106a* knockdown did not abolish LPS-induced pro-inflammatory cytokine production, suggesting that Tmem106a activity might be not associated with LPS-mediated response. Taken together Tmem106a expression is required for induction of pro-inflammatory cytokines by anti-Tmem106a in mouse macrophages, indicating the specificity of action of anti-Tmem106a.

### Anti-Tmem106a activates the MAPK pathway

**Figure 5:**
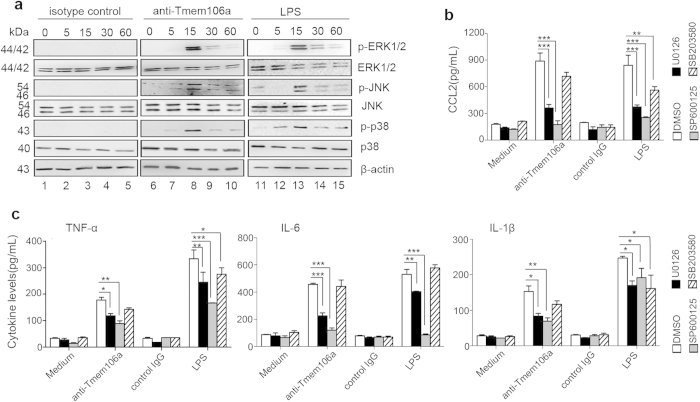
Anti-Tmem106a activates MAPK signals. (**a**) Mouse macrophages were serum-starved for 24 h and thereafter exposed to isotype IgG (lanes 1–5), anti-Tmem106a (lanes 6–10) or LPS (lanes 11–15) for the periods of time indicated. Then the levels of phosphorylated MAPK and total MAPK were assessed by Western blot with specific antibodies. Full-size images of western blots are shown in Supplementary Figure S7. (**b** and **c**) Mouse thioglycollate-elicited peritoneal macrophages were pretreated with DMSO, U0126, SP600125, SB203580 for 1 h followed by stimulation with anti-tmem106a, isotype IgG and LPS for 24 h, The levels of CCL2, TNF-α, IL-6 and IL-1β in the cultural supernatants were determined by ELISA. The results are expressed as mean ± SD from three independent experiments (*p < 0.05, **p < 0.01, ***p < 0.001).

We next explored the possible mechanism underlying macrophage activation by anti-Tmem106a. Macrophages were exposed to serum free medium for 24 h, and then incubated with anti-Tmem106a, isotype control IgG or LPS for 0, 5, 15, 30 or 60 min. Then, total MAPKs and phosphorylated MAPKs were assessed by immunoblotting. Compared with the isotype control IgG group, treatment with LPS and anti-Tmem106a resulted in an increase in activation of MAPK. In the LPS treated group, phosphorylation of ERK-1/2, JNK, and p38 MAPK peaked after 15 min, and then declined. Interestingly, increased phosphorylation of ERK-1/2, JNK, and p38 MAPK levels was also detected at 15 min after anti-Tmem106a stimulation, and disappeared after approximately 60 min (Fig. 5a).

To further analyze whether MAPK signals are involved in anti-Tmem106a-induced macrophage activation, U0126 (a MEK1/2 inhibitor), SP600125 (a JNK inhibitor) and SB203580 (a p38 MARK inhibitor) were used to inhibit the activities of ERK1/2, JNK and p38 MARK, respectively. Mouse thioglycollate-elicited peritoneal macrophages were pretreated with DMSO control, U0126 (10 μM), SP600125 (50 μM) or SB203580 (10 μM) for 1 h, respectively, followed by stimulation with or without of anti-Tmem106a, isotype control IgG or LPS for 24 h. The concentrations of CCL2, TNF-α, IL-6 and IL-1β in the cultural supernatants were determined by ELISA analysis. As shown in Fig. 5b, treatment with indicated MAPK inhibitors significantly reduced the secretion of CCL2 in macrophages stimulated by LPS. In anti-Tmem106a treated macrophages, both U0126 and SP600125 markedly decreased the levels of CCL2, but little for SB203580. As a negative control, isotype control IgG treated cells had no any effects. Simultaneously, under the same treatment condition, we assessed the levels of pro-inflammatory cytokines. Figure 5c shown that treatment with U0126 or SP600125 markedly inhibited the production of TNF-α, IL-6 and IL-1β caused by anti-Tmem106a. SB203580 had no obvious effect on the cells. As positive control, that treatment with MAPK inhibitors significantly reduced the secretion of TNF-α, IL-6 and IL-1β stimulated by LPS. This was expected because MAPK signals play essential role in TLR mediated inflammatory responses. These data indicate that anti-Tmem106a-mediated macrophage activation was partially inhibited by inhibition of ERK1/2 and JNK activities.

### Anti-Tmem106A promotes activation of the NF-κB and STAT1 pathways

**Figure 6:**
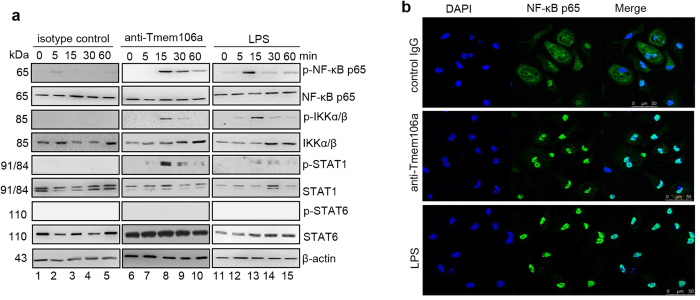
Anti-Tmem106a induces activation of NF-κB and STAT1 signals. (**a**) Mouse macrophages were serum-starved for 24 h and thereafter exposed to isotype IgG (lanes 1–5), anti-Tmem106a (lanes 6–10) or LPS (lanes 11–15) for the periods of time indicated. Then, the levels of total and phosphorylated NF-κB p65, IKKα/β, STAT1 and STAT6 were assessed by western blot with specific antibodies. Full-size images of western blots are shown in Supplementary Figure S8. (**b**) For confocal laser-scanning microscopy, viable naïve peritoneal macrophages on glass slides were treated with control IgG, anti-Tmem106a or LPS for 30 min, followed by treatment with FITC-labeled goat anti-mouse NF-κB p65 antibody for 12 h at 4 °C and DAPI stainning. The slides were observed under a Leica confocal.

NF-κB and JAK/STATs also play crucial roles in immune responses, and are important transcriptional regulators of inflammatory cytokines. To determine if these signaling pathways were involved in macrophage activation induced by anti-Tmem106a, phosphorylated NF-κB or JAK/STAT were determined by western blot with appropriate antibodies. Data from experiments indicated that there was no obvious phosphorylation in control IgG treated cells (Fig. 6a). In the positive control, LPS-stimulated macrophages displayed high levels of phosphorylated NF-κB p65, IKKα/β and p-STAT1, but not p-STAT6 (Fig. 6a). A similar effect was detected in anti-Tmem106a treated group (Fig. 6a).

Since nuclear translocation and activation of NF-κB p65 is preceded by the degradation of IκB and activation of IKKα/β, we next examined the distribution of the cytosolic NF-κB p65 subunit in mouse macrophages by confocal microscopy. Figure 6b shows that, like LPS, anti-Tmem106a markedly induced nuclear translocation of the NF-κB p65 subunit compared with isotype control IgG (Fig. 6b).

## Discussion

The human *TMEM106A* gene was first discovered and cloned by our laboratory^14^. Previous investigations indicated that human *TMEM106A* is a type II membrane protein, downregulated or silenced by promoter region hypermethylation in gastric cancer cell lines and primary gastric cancer tissues, but expressed in normal gastric tissues. Overexpression of TMEM106A suppressed cell growth and induced apoptosis in GC cell lines, and retarded the growth of xenografts in nude mice. We proposed that *TMEM106A* is a novel functional tumor suppressor in gastric carcinogenesis. In the present study, we successfully characterized the immunobiological functions of mouse Tmem106a, for which there are no previous reports. Protein structure analysis and electronic expression profiling indicated that Tmem106a is also a type II transmembrane protein and is expressed in mouse macrophages. Flow cytometry and confocal expression analysis confirmed these bioinformatic predictions (Fig. 2).

Macrophages express many receptors that mediate their diverse functions such as opsonic receptors including complement receptors and Fc receptors^23^,^24^, and non-opsonic receptors including Toll-like receptors (TLR)^25^, scavenger receptors and C-type lectins^26^,^27^. Cytosolic viruses and bacterial products are recognized by the NOD-like receptors (NLR) and RIG-like receptors (RLR)^28^. TLRs activate inflammatory gene expression by inducing activation of NF-κB, IFN-regulatory factors (IRF) and MAPKs. Fc receptors have either an inhibitory or activatory effect on NF-κB induction through ITIM or ITAM, respectively^23^,^24^. Scavenger receptors and lectins have been shown to induce NF-κB. NLR induces NF-κB and caspase-1. RLR either induces NF-κB and IRF, or induces caspase-1-mediated apoptosis^29^. As shown in this work, membrane-expressed Tmem106a could induce activation of the MAPK and NF-κB pathway in mouse macrophages. As a transmembrane protein, Tmem106a could be an unknown receptor or a new type of receptor. More works are necessary to determine whether or not the function of Tmem106a involves collaboration with TLR or CLR.

As an effective mouse macrophage activator, Tmem106a is able to upregulate the expression of pro-inflammatory cytokines IL-1β, TNF-α, IL-6 and chemokine CCL2, induce MAPK phosphorylation and activate the NF-κB pathway in mouse macrophages. Moreover, it is also capable of inducing NO production, STAT-1 phosphorylation, and expression of CD80, CD86 and MHC-II. IL-1β, TNF-α and IL-6 are typical cytokines of M1 macrophages. The activation of NF-κB, specifically subunit p65, is a hallmark of classical macrophage activation^30^. The balance between activation of STAT1 and STAT3/STAT6 finely regulates macrophage polarization and activity. A predominance of NF-κB and STAT1 activation promotes M1 macrophage polarization, resulting in cytotoxic and inflammatory functions such as NO production and expression of MHC-II^31^. Therefore, Tmem106a may be a newly triggering signal that enhances macrophage polarized sensitivity toward to M1.

Activation of Tmem106a on cell surfaces promotes high TNF-α, IL-1β, IL-6 levels and low IL-10 production in macrophages, and may enhance microbicidal effector functions through the induction of inducible nitric oxide synthase (iNOS) and NO, which participate in the activation of oxidative processes that contribute to the killing of invading organisms. Typical M1 macrophages usually exhibit a pro-inflammatory phenotype in response to microbial challenge, and then kill invading organisms. Therefore, it is worth considering that Tmem106a might make macrophages efficient in killing bacteria, viruses, parasites and fungi, and therefore that it plays a critical role in inflammation and host defense. These will be the emphases of our future studies.

The effect of macrophages in tumor progression has been shown to be double-edged, since these cells can both promote tumor rejection (M1 macrophages) and stimulate tumor growth (M2 macrophages). Pro-inflammatory M1 macrophages have the potential to exhibit antitumor activity and to elicit tumor tissue disruption^2^,^32^,^33^,^34^; they are thought to contribute to the T cell–mediated elimination and equilibrium phases during tumor progression^35^. Infiltrating M1 macrophages in tumor tissue were significantly associated with an improved prognosis or patient survival in colorectal carcinoma (CRC), non-small cell lung cancer, and liver cancer^36^,^37^,^38^,^39^,^40^. In this work, Tmem106a was shown to be a very effective M1 macrophage agonist, so activation of macrophages through the Tmem106a pathway may be a new way to fight tumors.

Taken together, our results indicate that mouse Tmem106a, a transmembrane protein, is a potent immunostimulatory molecule for macrophages via the MAPK, NF-κB and STAT-1 pathways. Since Tmem106a may be a novel receptor on macrophages, the trigger of this receptor, the ligand of Tmem106a, must play a crucial role in macrophage activation. Further studies will clarify what is this ligand, and how it affects the macrophage function.

## Methods

Female C57BL/6 (H-2^b^) mice, 8–10 weeks of age, were provided by Unilihua Bioscience Center, Beijing, China. All animals were maintained in the specific pathogen free (SPF) laboratory of the Experimental Animal Division of Peking University Health Science Center. All animal experiments were approved by the Beijing Experimental Animal Management Authority, Beijing, China. All methods used in this study were carried out in accordance with the approved guidelines.

### Antibodies, reagents

Polyclonal antibodies against mouse Tmem106a were prepared by immunizing rabbits with chemically synthesized Tmem106a peptides (Figure S1, boxed sequences), purified by peptide affinity chromatography with CNBr-activated Sepharose^TM^ 4 Fast Flow (GE Healthcare, 17-0981-01), according to the manufacturer’s instructions. Other primary antibodies used in this study were: anti-NF-κB (p65), ERK-1/2, JNK, p38 MAPK, phosphor (p)-ERK-1/2 (Thr202/Tyr204), p-JNK (Thr183/Tyr185), p-38MAPK (Thr180/Tyr182), p-NF-κB (p65), Stat Antibody Sampler Kit and p-Stat Antibody Sampler Kit (all purchased from Cell Signaling Technology, Beverly, MA, USA). U0126, SB203580 and SP600125 were also from Cell Signaling Technology. Recombinant mouse IL-4 was from R&D Systems (MN, USA). Horseradish peroxidase or FITC-conjugated anti-rabbit-IgG antibodies were from Southern Biotechnology Associates Inc. (Birmingham, AL, USA).

The followings are the sequences of double-stranded siRNAs against *Tmem106a*, which were designed and chemically synthesized by Genechem Corporation (Shanghai, China): *siTmem106a*-1: 5'-GCA CAC UCC UGU AUG CCU UTT-3'; *siTmem106a* -2: 5'-GGU UGC UCU CAU CCC UUA UTT-3'. The non-silencing control siRNA was confirmed to have no matches with the complete mouse genome by a BLAST search (www.ncbi.nlm.nih.gov).

### Preparation of mouse peritoneal macrophages

In order to obtain mouse peritoneal macrophages, C57BL/6 mice were treated with or without 3% thioglycollate 3 days before they were sacrificed by cervical amputation. Peritoneal macrophages were harvested by peritoneal lavage using ice-cold Ca^2+^ and Mg^2+^ -free PBS. For time-response assays, the macrophages were exposed to serum free medium for 24 h to obtain quiescent cells and then cultured with or without anti-Tmem106a, isotype control IgG, or LPS, for various times from 0 to 60 min.

### Cell culture, transfections and treatments

Tumor cell lines Raw264.7, NIH-3T3, P815, Sp2/0, HeLa, MDA-231 and THP-1 were from American Type Culture Collection, USA, and were maintained in our laboratory. All cells were cultured in RPMI-1640 (Hyclone, USA) supplemented with 10% (v/v) FCS (Hyclone), penicillin/streptomycin (100 U/mL), L-glutamine (2 mM) and 2-ME (5 × 10^−5^ M).

Mouse preritoneal macrophages were transfected with 600 pmol of siRNA using Lipofectamine 2000 reagent according to the manufacturer’s instructions (Invitrogen, USA). Briefly, 600 pmol of siRNA and 30 μL of Lipofectamine 2000 were diluted with Opti-MEM to a final volume of 3 mL, and added to mouse macrophages grown to 60% confluence in 100 mm diameter plates. 6 h later, cells were washed and then cultured in fresh medium for 24 h. Cells were then washed and analyzed to determine the effectiveness and specificity of siRNA treatment against Tmem106a.

To determine the inhibition of the MAPK signaling pathway, mouse macrophages were pre-treated with 10 μM of U0126 or SB203580 (10 μM) or SP600125 (50 μM) for 1 h, followed by incubation with or without anti-Tmem106a, LPS or isotype control IgG control for 24 h, then harvested and analyzed.

### RT-PCR assay

Total cellular RNA samples from mouse tissues were extracted using the Trizol^TM^ Reagent (Invitrogen). RT-PCR was performed using the ThermoScript RT-PCR System (Invitrogen). Primers used for amplifying *Tmem106a* were: 5'- ATGGGTAAGGCAGTCTCC-3' (sense) and 5'-TGCTGGTGACCTGGCCTA-3' (antisense); *β-actin* primers were 5'-GTG GGG CGC CCC AGG CAC CA-3' (sense) and 5'-CTT CCT TAA TGT CAC GCA CGA T TTC-3' (antisense).

### Western blotting

Total protein from cells or mouse tissues was extracted using RIPA buffer. Following solubilization, proteins were quantified using a BCA protein quantification kit (Thermo Scientific, Rockford, IL, USA). Fifty micrograms of protein were loaded into each well and separated on a 10% SDS Tris-Glycine gel, then transferred onto polyvinylidene difluoride (PVDF) membranes (Invitrogen) at a constant current of 250 mA in transbuffer (50 mM Tris, pH 8.0, 0.192 M glycine, 20% (v/v) methanol), using a Bio-Rad Trans-Blot Cell (Bio-Rad, Hercules, CA, USA). The membranes were incubated for 1 h at room temperature in blocking buffer (TBS containing 5% non-fat milk), followed by an overnight incubation at 4 °C with appropriate primary antibodies in blocking buffer. After three washes with TBS containing 0.05% Tween 20, strips were incubated for 1 h with corresponding HRP-conjugated secondary antibodies. Protein-antibody complexes were visualized using an ECL detection system as recommended by the manufacturer (Applygen Technologies, Beijing, China). Data were recorded using a Bio-Rad Gel Doc 2000 system.

### Flow cytometry, Immunofluorescence and confocal microscopy

RAW264.7 cells or mouse macrophages were centrifuged and the cell pellets (1 × 10^6^/tube) were incubated with FITC-conjugated anti-Tmem106a or isotype control IgG for 1 h at 4 °C. The cell surface expression of mouse Tmem106a was analyzed by flow cytometry on a FACS Caliber. In some experiments, the mouse macrophages were treated with anti-Tmem106a, LPS or isotype IgG control for 20 h, before staining with FITC-conjugated anti-mouse CD80, PE-conjugated mouse CD86, CD69 or MHC class II (Ia/Ie) for 1 h at 4 °C, followed by flow cytometric analysis.

For confocal microscopy observation, cells were fixed using 4% paraformaldehyde in PBS. Blocking was performed using 3% BSA diluted in PBS for 30 min at room temperature. Cells then were incubated with primary antibodies overnight at 4 °C, followed by incubation with corresponding fluorescein-conjugated secondary antibodies. Nuclei were stained with DAPI (Biostatus Limited). The results were observed with a Leica SP5 confocal microscope (DMRE, Leica).

### Detection of cytokines and nitric oxide (NO)

Different cells were cultured with or without anti-Tmem106a (10 μg/mL), isotype control IgG (10 μg/mL) or LPS (5 μg/mL) in 24-well plates for 40 h. In some inhibition assays, cells were also pretreated with of U0126 (10 μM), SB203580 (10 μM), SP600125 (50 μM) or siRNAs before reagent stimulation. Concentrations of TNF-α, IL-6, IL-1β and mononuclear chemoattractant protein-1 (CCL2/MCP-1) in supernatants were determined using ELISA kits from eBioscience (San Diego, CA, USA) following the manufacturer’s protocol.

NO release was evaluated by measuring nitrite levels, according to the method described by the kit manufacturer (Madison, Wisconsin, USA). Cells were cultured with or without anti-Tmem106a (10 μg/mL), control IgG (10 μg/mL) or LPS (5 μg/mL) for 24 h. The incubation medium (100 μL) was mixed with 10 μL of Griess reagent (0.1% N-1-naphthylediamine, 1% sulfanilamide in 5% H_3_PO_4_) and optical density was measured at 540 nm. The amount of nitrite in the incubation medium was calculated using sodium nitrite as a standard.

### Statistical analysis

All experiments described here have been repeated at least three times. Results are presented as mean ± standard deviation (SD). Comparison of the data was performed using Student’s *t* test. Significance was defined as *p* < 0.05. Statistical analysis was performed using SPSS software.

## Additional Information

**How to cite this article**: Dai, H. *et al.* Transmembrane protein 106a activates mouse peritoneal macrophages via the MAPK and NF-κB signaling pathways. *Sci. Rep.* **5**, 12461; doi: 10.1038/srep12461 (2015).

## Supplementary Material

Supplementary Information
