# Supplementary material for: Transmembrane protein 106a activates mouse peritoneal macrophages via the MAPK and NF-κB signaling pathways
Source: Sci Rep. 2015 Jul 28;5:12461. doi: 10.1038/srep12461 (PMC4516968; doi:10.1038/srep12461)

Supplementary Information:

Transmembrane protein 106A activates mouse peritoneal macrophages via the MAPK and NF-κB signaling pathways

Hui Dai[1], Dong Xu[1,2], Jing Su[3], Jingyuan Jang[1], Yingyu Chen[1,2]

[1]Key Laboratory of Medical Immunology, Ministry of Health, Peking University Health Science Center, Beijing, China

[2] Center for Human Disease Genomics, Peking University, Beijing, China.

[3] Department of Pathology, School of Basic Medical Sciences, Peking University Health Science Center, Beijing, China

*Corresponding Email: yingyu_chen@bjmu.edu.cn

Figure S1

```
1     ggggccgagcccggcttggactccgagcttgtgttaaatgtgcagctcccgcagtttcgattttgctgagggcgttgtcacctgaggccc
91    ttctccccaaaggagcattccagaaaacccaatcttggagctgagcagagcctggaatcccagacctaaaccagtgaaaaattccgctgg
181   actatgggtaaggcagtctcccagctcacctctcggaaggatgaggacaagcctatcctacctgataacccagccatggccagccaggct
         M  G  K  A  V  S  Q  L  T  S  R  K  D  E  D  K  P  I  L  P  D  N  P  A  M  A  S  Q  A
271   gccaactacttcagcactggtagcagcaagccagcacactcctgtatgccttatgaaaaggctgctagttccagctttgtgacttgtcct
       A  N  Y  F  S  T  G  S  S  K  P  A  H  S  C  M  P  Y  E  K  A  A  S  S  S  F  V  T  C  P
361   acctgccaaggcaatggggagatcccccaagagcaagagaagcagctggttgctctcatcccttatggagaccaaaggctgaagcccaga
       T  C  Q  G  N  G  E  I  P  Q  E  Q  E  K  Q  L  V  A  L  I  P  Y  G  D  Q  R  L  K  P  R
451   cgcacgaagctctttgtgttcctgtcggtggccatctgcctgctgatttctccctcaccatctttttcctgtatccccggcccattgct
       R  T  K  L  F  V  F  L  S  V  A  I  C  L  L  I  F  S  L  T  I  F  F  L  Y  P  R  P  I  A
541   gtgcgtcctgtcggcctcaactcttccacagtaacctttgaagacgctcacgtacagctcaacacgacgaatgtcctgaacatcttcaac
       V  R  P  V  G  L  N  S  S  T  V  T  F  E  D  A  H  V  Q  L  N  T  T  N  V  L  N  I  F  N
631   agcaacttctatcccatcacagtgacacagctgacggccgaggtgctccaccaggcctctgtggtaggccaggtcaccagcagcctccgc
      S  N  F  Y  P  I  T  V  T  Q  L  T  A  E  V  L  H  Q  A  S  V  V  G  Q  V  T  S  S  L  R
721   ctgcacatcggcccattggccagtgagcagatgccttacgaagttgccagcaggatttggatgaaaacacatacaaaatctgtacatgg
       L  H  I  G  P  L  A  S  E  Q  M  P  Y  E  V  A  S [R  I  L  D  E  N  T  Y  K  I  C] T  W
811   ccgaaaatcagagtccaccatattcttttgaatatccaggttctctgacctgctccttcctaagccatccgcaacagctgcccttcgag
       P  K  I  R  V  H  H  I  L  L  N  I  Q  G  S  L  T  C  S  F  L  S  H  P  Q  Q  L  P  F  E
901   agctttgaatatgtagactgcagagaaaacatgtccctgccccacctggagctgccccgcccagcctgatctgtctgccgcccctggact
       S  F  E  Y  V  D  C [R  E  N  M  S  L  P  H  L  E  L  P  R  P  A] *
991   ccaggcacctgcaagctgatctgcagctcagctccgtggagctcaaggaaagactgtagggactttgttgaaggaaaagtcctgctttgg
1081  gccctgccttcccctaacaccctcagcacacagagcttctgtaacaatgtcatagtccctgagtcctcagacctccccactcccccacga
1171  gtcgcttctgatggatcttacccatggtctgtgtggggaggaacggctaccttggcgttgtcttgggactgagcatgttggttacccagc
1261  tccgccagggtgggtcttcaatcgcaacgaagagaaagctgtagttttatctagactttgcagcatggatgaatctttttcaagctctcc
1351  tcccagtaataagtggctttggggatatccagtgctgtttccaaggtctccttgggtgactataaatgttctcctgtactgtcttgcttt
1441  gatcatgtgataatgagggtaatagactacttctctcagtaaatggtagacaataaggtcctcatcctcccaggttcggaatactatccc
1531  tggcttccaggaagggagatttacaccatctaaactcttctctatagactggctccaatgctatcttcacagcagaaaccaaggagctga
1621  ggagaaaccatcgggtgtaccctagagcaaaggcagagccagttgcaaagatatttattaaaggaaagtttctaagcccaccatccagag
1711  cccatgtgagtgcacacaggtaagacaggcagtagagcaggcccaggggcagtgttcagaaagcatgaaggacataagctgttgatacca
1801  ggccaaagctgtggggctctcatggattctctgcatcaggcccaccctctgctgtcctctgggatgtgactgaagagtgacagattcacc
1891  agaatttccttccttgcctgtcactacactgacgccaaaccacaggtaacccccagttttctactggactccaggtggagatatgatgtc
1981  tgttgccggagagcagaaatgtgtgttctctaaagctagctagcctctgccctccagacaggtgcaaaggcgaagggcggtgtggttggg
2071  gaaaattgaatttgaagtgagaagaatgcaaggaaatggtggtaactgagctgggtaactagcctggggctacacagagggagaggaagt
2161  cttgtctgcaccaagttttccctcctgggtagccagggctaaggtgtacaaaccaaaggagacccagggaagtcaagctgagattagagt
2251  gtataaaccatcaaatgtaaaagatttctaacaataaattgttctttagcc
```

Figure S1. Nucleotide sequence and predicted amino acid sequence of mouse Tmem106a. The start and stop codons are underlined, and the putative TM domain (amino acid residues from 93 to 115) is indicated with a broken line.

Figure S2

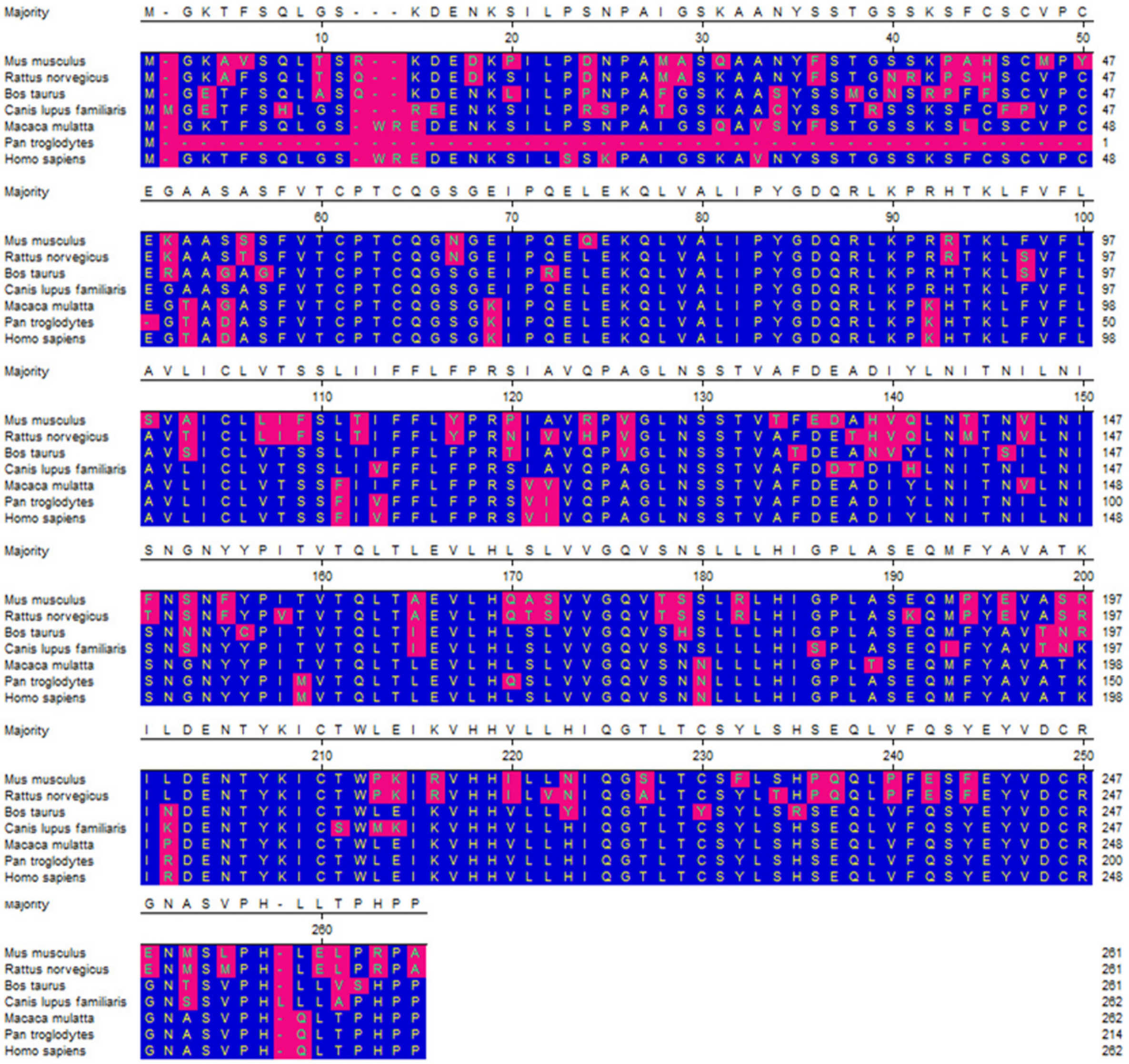

Figure S2. Sequence alignment of TMEM106A proteins from different organisms. Different colors indicate different identity levels.

Figure S3

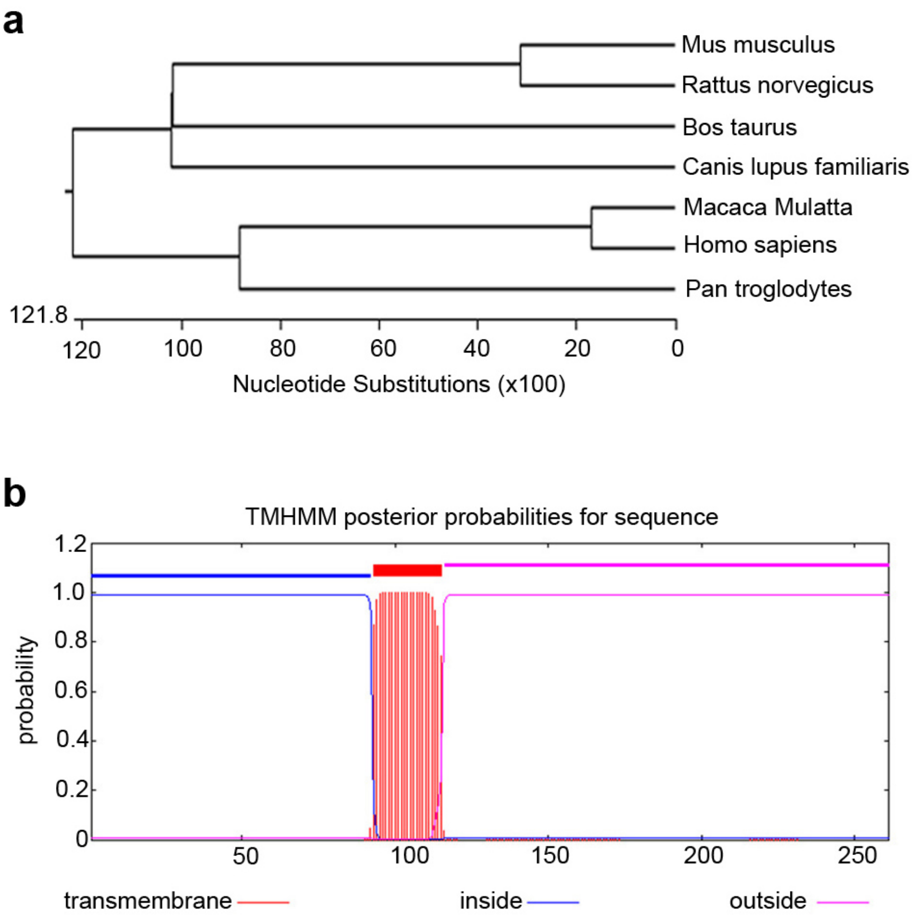

Figure S3. **(a)** Phylogenetic analysis of *TMEM106A* genes. (**b**) Transmembrane (TM) analysis of mouse Tmem106a protein.

Figure S4 Full-size images of RT-PCR and western blots for Tmem106a and β-actin

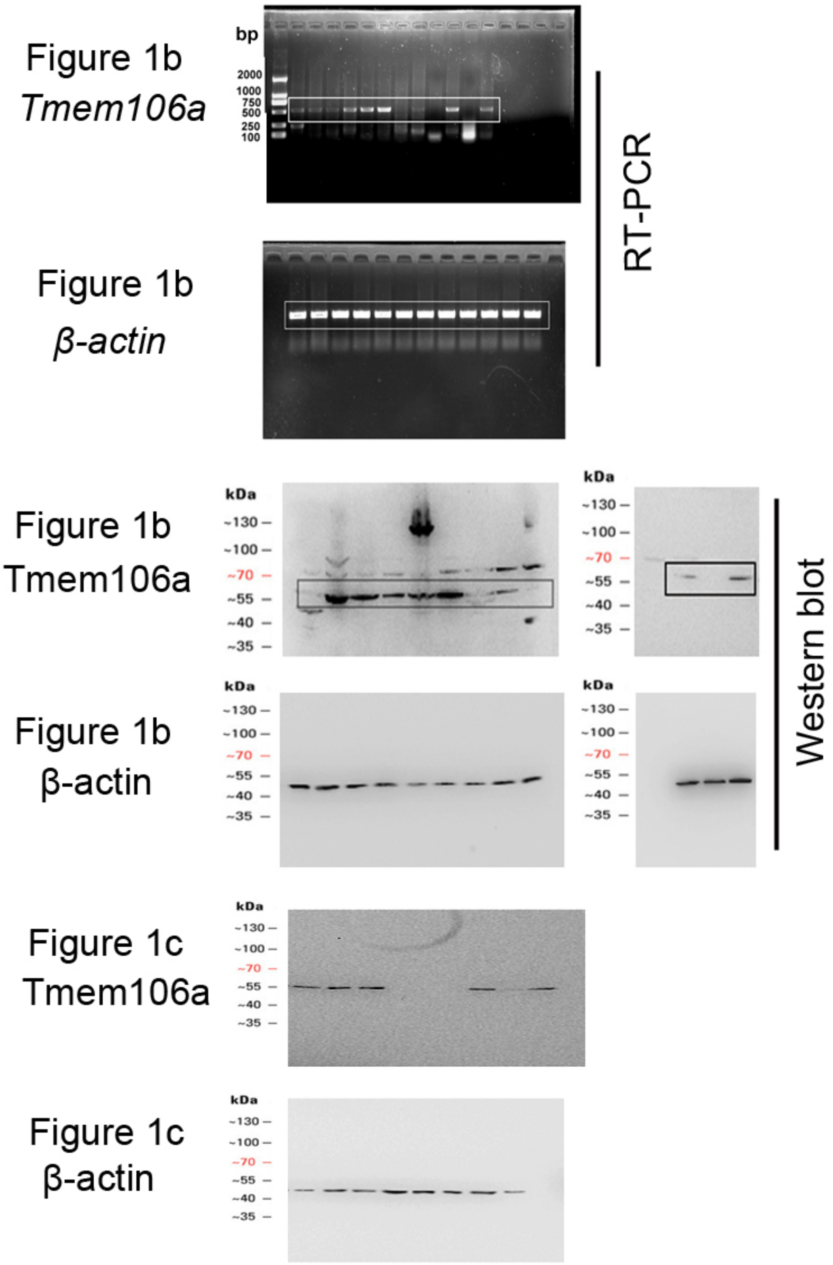

Figure S5 Full-size images of western blots for iNOS, arginase and β-actin

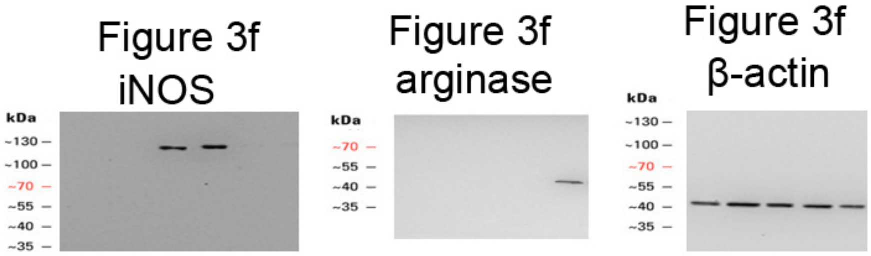

Figure S6 Full-size images of western blots for Tmem106a and β-actin

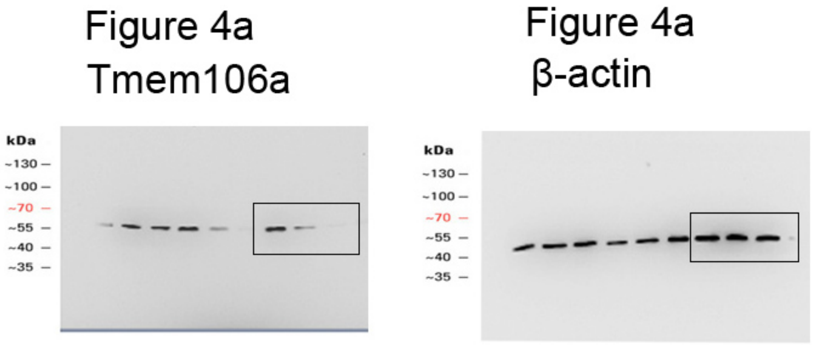

Figure S7 Full-size images of western blots for ERK, p38, JNK and β-actin

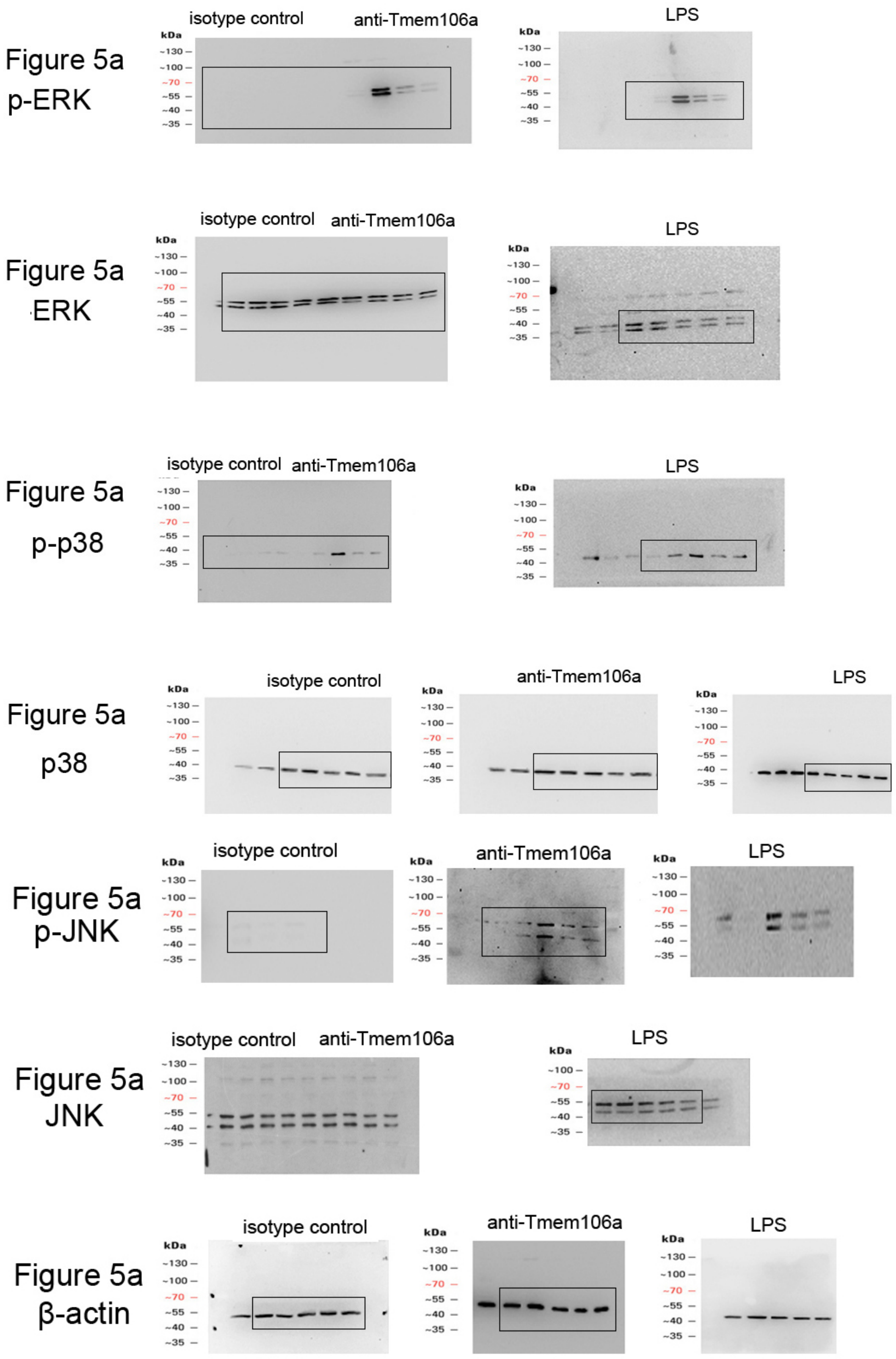

Figure S8 Full-size images of western blots for NF-κB p65, IKKα/β, STAT1, STAT6 and β-actin

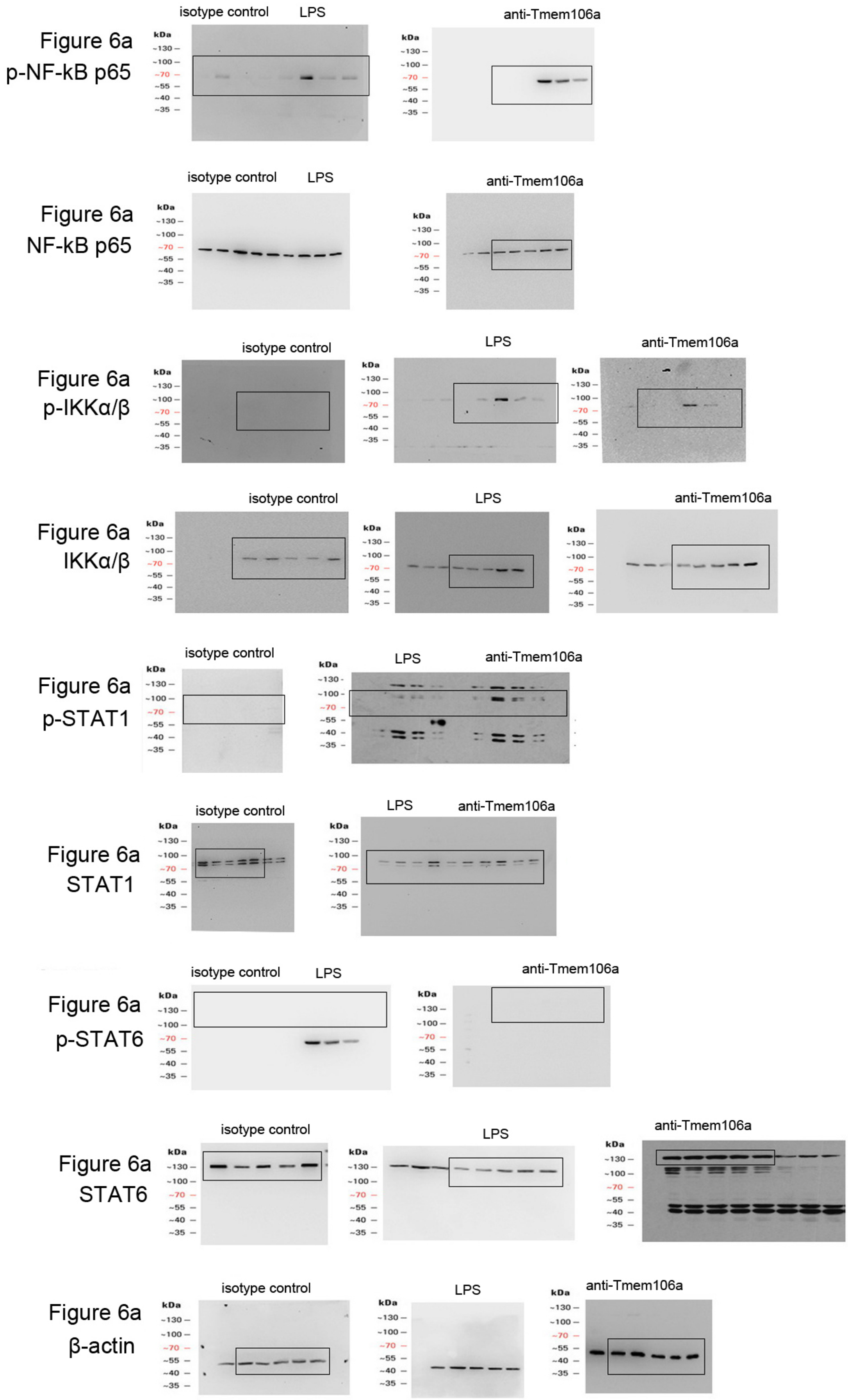

Supplement: Supplementary Information [file srep12461-s1.pdf]
